# TRPA1 and TRPM8 in Allergic Rhinitis and Chronic Rhinosinusitis: Emerging Role in Neuroimmune Inflammation

**DOI:** 10.3390/biomedicines14051015

**Published:** 2026-04-30

**Authors:** Tianhui Kang, Zijun Qiu, Surita Aodeng, Yuzhuo Liu, Zhenzhen Zhu, Wei Lv

**Affiliations:** Department of Otolaryngology-Head and Neck Surgery, Peking Union Medical College Hospital, Chinese Academy of Medical Sciences and Peking Union Medical College, Beijing 100730, China; kangth0721@126.com (T.K.); qzjhjqw1109@163.com (Z.Q.); aodengsurita@pumch.cn (S.A.); lyzminminliu@163.com (Y.L.)

**Keywords:** allergic rhinitis, chronic rhinosinusitis, nasal hyperreactivity, neuroimmune, TRPA1, TRPM8

## Abstract

Nasal hyperreactivity (NHR) is a core symptom of allergic rhinitis (AR) and chronic rhinosinusitis (CRS), frequently induced by cold stimuli. Accumulating evidence indicates that NHR is largely mediated by neuroimmune mechanisms rather than classical allergen-driven inflammation alone. Among the molecular sensors involved, the cold-sensitive transient receptor potential channels transient receptor potential ankyrin 1 (TRPA1) and transient receptor potential melastatin 8 (TRPM8) have emerged as key regulators linking environmental cold exposure to sensory nerve activation, neuropeptide release, and immune modulation. This review systematically summarizes the expression, functions, and neuroimmune mechanisms mediated by TRPA1 and TRPM8 in AR and CRS, with a particular focus on their roles in NHR. Furthermore, it discusses the therapeutic potential of targeting these channels to alleviate neurogenic inflammation and refractory nasal symptoms, aiming to provide new perspectives for understanding disease mechanisms and developing precise treatments.

## 1. Introduction

Allergic rhinitis (AR) and chronic rhinosinusitis (CRS) are highly prevalent chronic inflammatory diseases of the upper airway with marked geographic heterogeneity and a continuously increasing global incidence [1,2,3]. Globally, the prevalence of AR ranges from 10 to 30% in the general population, with substantial variations across continents: 23–30% in Europe, 12–30% in North America, 15–25% in Asia, and 3.6–22.8% in Africa [3]. The global pooled prevalence of CRS is 8.71%, with regional differences observed across European, Asian, and American populations [2,4]. Clinically, they share common clinical manifestations, including nasal obstruction, rhinorrhea, sneezing, and nasal hyperreactivity (NHR), and impose substantial personal and socioeconomic burdens [5]. Despite advances in pharmacological and surgical treatments, a considerable proportion of patients continue to experience persistent or refractory symptoms.

NHR, a hallmark feature of both AR and CRS, refers to the induction of symptoms like rhinorrhea, sneezing, nasal itching, or obstruction upon exposure to specific environmental stimuli such as changes in temperature or humidity [6,7]. Clinically, NHR can be objectively assessed by a ≥20% reduction in peak nasal inspiratory flow (PNIF) following cold-dry air (CDA) provocation [8]. Cold-induced NHR shows strict climate and population specificity: it is highly prevalent in temperate and cold climate regions, affecting approximately 70% of AR patients upon CDA exposure [9]. In contrast, tropical regions exhibit high AR prevalence but low cold-induced NHR, as cold is not a dominant environmental trigger in this setting [3]. NHR also occurs in CRS patients, though with lower prevalence compared to AR [5]. Importantly, NHR frequently occurs independently of allergen exposure or overt inflammatory exacerbation, suggesting that mechanisms beyond classical immune pathways contribute to symptom generation [5].

In recent years, increasing attention has been directed toward the role of the sensory nervous system in upper airway inflammation. Sensory nerve fibers densely innervate the nasal mucosa and are strategically positioned to detect physical and chemical environmental changes [5]. Rather than serving solely as passive conduits for sensory perception, these neurons actively participate in immune regulation by releasing neuropeptides and interacting with epithelial and immune cells [10]. Through these neuroimmune interactions, sensory nerves contribute to vascular permeability, glandular secretion, immune cell recruitment, and the amplification of local inflammatory responses, thereby playing a pivotal role in both symptom initiation and disease persistence [11].

At the molecular level, transient receptor potential (TRP) channels have emerged as key sensors that enable sensory neurons to translate environmental stimuli into biological signals. Among them, transient receptor potential ankyrin 1 (TRPA1) and transient receptor potential melastatin 8 (TRPM8) are the principal cold-sensitive ion channels expressed on sensory nerve endings within the nasal mucosa [12,13].

Activation of TRPA1 and TRPM8 by cold stimulation induces neuronal depolarization and triggers the release of neuropeptides such as calcitonin gene-related peptide (CGRP) and substance P (SP) [14,15]. These mediators, in turn, initiate neurogenic inflammation, defined as a localized inflammatory response driven by neuropeptide release from activated sensory nerves via local or retrograde signaling pathways [16,17]. Through this mechanism, cold exposure can directly provoke nasal symptoms and inflammatory responses, independent of classical allergen-driven immune activation. By functioning as molecular interfaces between environmental cold stimuli and neuroimmune signaling, TRPA1 and TRPM8 are increasingly recognized as central regulators linking cold exposure to NHR in AR and CRS [18].

Currently, systematic elaboration of the specific neuroimmune mechanisms of TRPA1 and TRPM8 in AR and CRS remains limited. Integrating recent research findings, this review comprehensively summarizes the functions and regulatory mechanisms of these two channels in the neuroimmune network of AR and CRS, aiming to provide a theoretical basis for revealing the pathological essence of the diseases and developing novel therapeutic strategies.

## 2. Cold-Sensitive TRP Channels as Molecular Sensors in the Nasal Mucosa

The TRP channels form a large superfamily of non-selective cation channels that sense physical and chemical stimuli (temperature, mechanical force, endogenous/exogenous ligands) [18,19]. TRPA1 belongs to the ankyrin subfamily, and TRPM8 belongs to the melastatin subfamily, both defined as thermosensitive transient receptor potential channels (thermoTRPs) for their inherent temperature sensitivity [12,13]. These channels are evolutionarily conserved across mammals, with highly homologous functional domains and conserved roles in thermosensation and inflammation [20,21,22].

Structurally, both channels assemble as homotetramers, with each monomer containing six transmembrane (S1–S6) helices and a pore-forming loop between S5 and S6 [12,23]. TRPA1 has an extra-long N-terminus with 14–18 ankyrin repeats for protein interaction and channel sensitization. TRPM8 has a short N-terminus and a long C-terminus with phospholipid phosphatidylinositol 4,5-bisphosphate (PI(4,5)P_2_)-binding and phosphorylation sites that control activation and desensitization [23,24]. These structural features define their distinct activation traits and druggability for therapy [22].

TRPA1 is widely distributed in sensory neurons, including dorsal root ganglia and trigeminal ganglia, as well as in non-neuronal cells such as epithelial cells, mast cells, endothelial cells, and fibroblasts, with particularly high expression in mucosal tissues of the respiratory tract [12]. TRPA1 can be activated by diverse exogenous compounds and endogenous inflammatory mediators [25]. Intense cold stimulation (temperature < 17 °C) also activates TRPA1, accompanied by upregulation of its expression level. TRPA1 gene-knockout mice exhibit significantly impaired responses to noxious cold stimuli, confirming its crucial role in cold perception [17,20,26]. In the respiratory tract, activation of TRPA1 induces nasal vasomotor modulation and airway smooth muscle contraction, contributing to the development of airway hyperreactivity [19,27,28,29].

TRPM8 is another major cold thermoreceptor, primarily located in primary afferent neurons of the trigeminal and dorsal root ganglia, and localized within the nasal epithelium, mucosal glands, and perivascular regions. In addition to being activated by low temperatures (15–28 °C), TRPM8 is sensitive to cooling compounds such as menthol [13]. TRPM8 plays a dominant role in cold perception, as demonstrated by the observation that TRPM8-deficient mice exhibit significant impairments in cold perception at temperatures between 15 and 28 °C, including attenuated behavioral responses to cold stimuli and compromised temperature discrimination ability [20,21,30].

Under physiological conditions, TRPM8 is involved in maintaining nasal homeostasis. When the nasal mucosa is exposed to cold air, TRPM8-mediated sensory pathways trigger local and central reflexes, leading to increased glandular secretion and rhinorrhea [31]. Furthermore, TRPM8 activity regulates nasal patency and airway resistance, thereby influencing the perception of nasal symptoms [32,33].

## 3. Sensory Nerve-Driven Neuroimmune Mechanisms in Nasal Hyperreactivity

Stimulation of sensory nerve endings in the nasal mucosa leads to Ca^2+^ influx, activating cold-sensitive receptors including TRPA1 and TRPM8. This activation promotes the release of various neuropeptides such as SP, CGRP, vasoactive intestinal peptide (VIP), neuromedin U (NMU), and neuropeptide Y (NPY) [22]. VIP regulates vasodilation and exerts anti-inflammatory effects via vasoactive intestinal peptide receptors 1 and 2 (VPAC1 and VPAC2); NMU promotes mast cell activation; NPY modulates vascular tone. Together, these neuropeptides directly induce vasodilation, increased vascular permeability, and glandular secretion, which are hallmark features of NHR [34,35,36].

**Figure 1:**
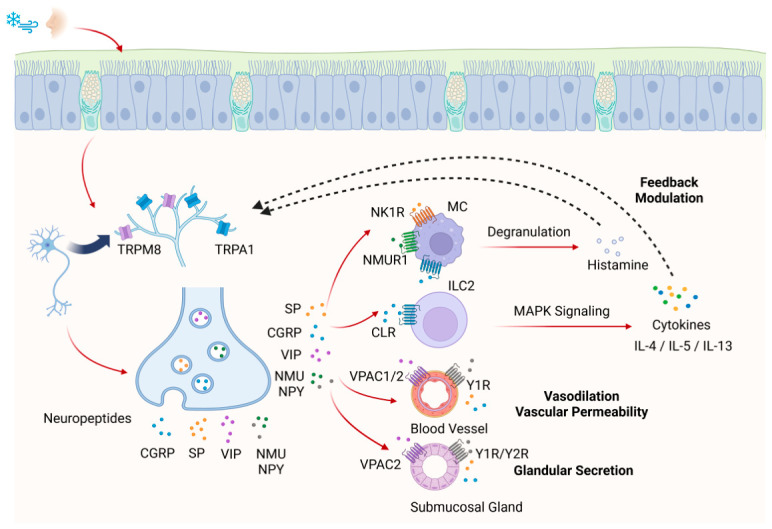
TRPA1/TRPM8-mediated neuroimmune interactions in nasal hyperreactivity. Cold stimulation of the nasal mucosa activates the cold-sensitive transient receptor potential channels, transient receptor potential ankyrin 1 (TRPA1) and transient receptor potential melastatin 8 (TRPM8) expressed on sensory nerve endings. Activation of these channels triggers neuronal depolarization and Ca^2+^ influx, leading to the release of neuropeptides including substance P (SP), calcitonin gene-related peptide (CGRP), vasoactive intestinal peptide (VIP), neuromedin U (NMU), and neuropeptide Y (NPY). SP and NMU activate mast cells (MCs) via neurokinin 1 receptor (NK1R) and neuromedin U receptor 1 (NMUR1), promoting degranulation and histamine release. CGRP acts on the calcitonin-like receptor (CLR) expressed on group 2 innate lymphoid cells (ILC2), stimulating mitogen-activated protein kinase (MAPK) signaling and subsequent type 2 cytokine production, which amplifies local inflammation. Collectively, these neuropeptides drive key vascular and mucosal effects, including vasodilation, increased vascular permeability, and submucosal glandular secretion. VIP and NPY mediate these responses via their specific receptors, vasoactive intestinal peptide receptors 1/2 (VPAC1/VPAC2) and neuropeptide Y receptors 1/2 (Y1R/Y2R), respectively. Meanwhile, immune cell-derived mediators, including histamine and type 2 cytokines, sensitize sensory neurons and enhance TRP channel activity, forming a positive feedback loop that perpetuates neurogenic inflammation and contributes to nasal hyperreactivity in allergic rhinitis and chronic rhinosinusitis. (Created in BioRender. Kang, T. (2026) https://BioRender.com/44zyp0m, accessed on 26 April 2026).

Beyond their direct effects on the nasal mucosa, neuropeptides orchestrate immune responses by activating key immune cells such as mast cells (MCs) and type 2 innate lymphoid cells (ILC2s), promoting the release of inflammatory mediators and thereby mediating neurogenic inflammation [17]. In turn, immune-derived mediators such as histamine and cytokines sensitize sensory neurons, forming a positive feedback loop that amplifies neurogenic inflammation [37]. Thus, cold thermoreceptors serve as a critical bridge, linking cold air stimulation of the nasal mucosa to the initiation of neuroimmune mechanisms (Figure 1). This bidirectional neuroimmune crosstalk provides a mechanistic explanation for the persistence of NHR even in the absence of active allergen exposure.

### 3.1. Neuroimmune Mechanisms of TRPA1 in AR

TRPA1 can be activated by exogenous cold stimuli as well as endogenous inflammatory mediators released by cells such as epithelial cells or mast cells. Neuronal TRPA1 activation mainly induces Ca^2+^-dependent exocytosis of CGRP, SP and other neuropeptides from nerve endings, initiating neurogenic inflammation [12]. Clinical studies indicate that cold exposure frequently induces AR exacerbations. AR patients show significantly upregulated TRPA1 mRNA expression in the nasal mucosa, correlating positively with clinical symptom severity [35,38]. Furthermore, AR patients exhibit heightened nasal responsiveness to TRPA1 agonists compared to healthy controls, suggesting enhanced TRPA1 sensitivity [39]. In murine models of AR and CRS, TRPA1 activation increases tissue levels of SP and CGRP, whereas TRPA1 antagonists effectively suppress neuropeptide release and associated neurogenic inflammation [40]. Clinical studies also report that higher baseline levels of SP and CGRP in nasal secretions of AR and CRS patients correlate with more intense responses to the CDA provocation test, although post-challenge neuropeptide levels remain stable [5]. These neuropeptides directly act on the nasal mucosa, inducing vasodilation and increased permeability [5].

Furthermore, the released neuropeptides interact with immune cells to regulate mast cell activation, ILC2 polarization, and upregulate inflammatory factors. In AR patients, SP preferentially promotes mast cell degranulation and histamine release via neurokinin-1 receptor (NK1R) activation, and NK1R expression is significantly upregulated in these patients [41]. Additionally, impairment of the “SP–Toll-like receptor (TLR) axis” represents a unique pathological hallmark in AR, which is characterized by delayed and prolonged TLR4 upregulation, reduced neutral endopeptidase expression, and abnormal NK1R levels. This dysfunction exacerbates innate immune dysregulation, increases infection susceptibility, and prolongs infection courses in AR [42]. CGRP primarily promotes mast cell degranulation by acting on CGRP receptors expressed on mast cells.

Meanwhile, CGRP exerts a dual regulatory effect on ILC2s. As key innate immune cells linking innate and adaptive immunity in lung tissue, ILC2s highly express the calcitonin receptor-like receptor (CLR). Studies in mice show that CGRP specifically binds to CLR on ILC2s, activating the mitogen-activated protein kinase (MAPK) signaling pathway within ILC2s. This leads to phosphorylation of the transcription factor GATA3, generating phosphorylated GATA3 (P-GATA3) [43]. P-GATA3 translocates to the nucleus, initiating the transcription of Th2 cytokines such as interleukin-4 (IL-4), IL-5, and IL-13, thereby promoting eosinophil infiltration and type 2 inflammation [34,35,44,45], indicating a pro-inflammatory role. However, mounting evidence has established a context-dependent anti-inflammatory function of CGRP in allergic airway inflammation. CGRP directly suppresses the proliferation of ILC2s and potently inhibits alarmin-driven production of type 2 cytokines, including IL-5 and IL-13 [46,47]. Mechanistically, CGRP signaling reshapes the transcriptional program of ILC2s toward a regulatory phenotype, thereby limiting excessive eosinophilic infiltration and tissue inflammation [47,48,49]. In vivo studies further confirm that CGRP deficiency or impaired CGRP receptor signaling results in enhanced ILC2 activation and aggravated type 2 inflammation, verifying its physiological anti-inflammatory and immune-homeostatic roles [46,47]. Thus, the pro-inflammatory or anti-inflammatory role of CGRP may depend on the specific inflammatory microenvironment.

This local axon reflex, whereby sensory nerves in the nasal mucosa release pro-inflammatory neuropeptides to generate inflammation, occurs without integration into the central nervous system (CNS). Furthermore, AR involves “central sensitization,” a phenomenon characterized by hyperresponsiveness of the CNS to nociceptive stimuli, leading to pathological amplification of responses to normal or subthreshold inputs and aberrant changes in neural output patterns [50], which further enhances neurogenic inflammation and hyperreactivity [51]. The existence of central sensitization has been confirmed in AR mice, where IL-4-mediated activation of the NLRP3 inflammasome in microglia induces central sensitization via the trigeminal nucleus caudalis (TNC), contributing to the pathogenesis of AR [52].

Mechanistically, epithelial TRPA1 activation is independent of neuronal signaling, which triggers Ca^2+^ influx and activates the calcineurin–nuclear factor of activated T cells (NFAT) pathway to directly drive the transcription and secretion of thymic stromal lymphopoietin (TSLP), IL-25 and IL-33, mediating epithelial-derived inflammatory amplification [53].

Conversely, various inflammatory mediators produced in the inflammatory microenvironment can regulate TRPA1 activity. Studies have shown that tumor necrosis factor-α (TNF-α) can upregulate the expression and function of TRPA1 in sensory neurons, enhance its current response, and promote neuropeptide release, forming an inflammatory amplification loop [37].

### 3.2. Neuroimmune Mechanisms of TRPA1 in Chronic Rhinosinusitis

Backaert et al. were the first to confirm that CRS patients develop NHR symptoms following CDA provocation, with the severity of nasal obstruction correlating with elevated levels of SP, CGRP, and histamine in nasal secretions [5]. These findings provide direct clinical evidence supporting the involvement of neuronal pathways in CRS-associated NHR. Consistent with this observation, TRPA1-driven neurogenic inflammation has been shown to modulate vasomotor tone, mucus secretion, and immune cell recruitment through the release of neuropeptides such as SP, CGRP, and VIP [36]. Distinct from AR, SP and CGRP in CRS do not induce impairment of the SP-TLR axis or drive dominant type 2 allergic inflammation [36,42]. In CRS, CGRP and SP act synergistically with VIP to control submucosal gland secretion and vascular permeability, which are closely associated with refractory nasal obstruction and rhinorrhea rather than type 2 allergic inflammation [54].

VIP is a potent vasodilatory and immunomodulatory neuropeptide that acts by binding to two G protein-coupled receptors, namely VPAC1 and VPAC2 [55]. Ligand binding triggers the Gαs–adenylyl cyclase–cyclic adenosine monophosphate (cAMP)–protein kinase A (PKA) signaling cascade, which mediates the core downstream effects of VIP, including suppression of pro-inflammatory cytokine production, inhibition of mast cell degranulation, and attenuation of eosinophil chemotaxis [55,56]. In the nasal mucosa of patients with chronic rhinosinusitis, both VIP and neuropeptide Y (NPY) are highly expressed in epithelial cells, eosinophils, and stromal cells, indicating a broad involvement of neuropeptide signaling in the local inflammatory microenvironment [36]. Beyond immune regulation, VIP modulates submucosal glandular secretion and maintains mucosal antimicrobial defense, and the homeostatic balance between VIP and NPY critically governs mucus production, innate immune defense, and the overall intensity of local inflammatory responses in CRS [54].

Conversely, immune cells can regulate neuronal activity through bidirectional neuroimmune crosstalk. For example, histamine released from activated mast cells enhances the sensitivity of trigeminal sensory neurons expressing TRPA1 to specific agonists in a histamine H1 receptor-dependent manner, thereby further amplifying neurogenic inflammation [5]. Despite these advances, current understanding of neuroimmune mechanisms in CRS remains limited. Key issues, including the precise molecular networks governing bidirectional neuroimmune interactions during disease progression and the long-term mechanisms underlying neuronal sensitization, remain to be fully elucidated and warrant further investigation.

### 3.3. Neuroimmune Mechanisms of TRPM8 in AR

As a key ion channel for cold stimulus perception, TRPM8 links cold stimulation to NHR in AR, serving as a critical molecular interface between environmental cold exposure and sensory nerve activation. Through TRPM8-mediated neuroimmune crosstalk, cold stimulation contributes to both the initiation and maintenance of nasal inflammation. Within the nasal mucosa, TRPM8 is primarily distributed in the submucosal region, with high enrichment around blood vessels, supporting its role in regulating vasomotor responses and sensory signal transmission [33].

Clinical studies demonstrate that TRPM8 expression in the nasal mucosa is negatively correlated with the magnitude of PNIF reduction during CDA provocation, while levels of neuropeptides such as SP and CGRP in nasal secretions positively correlate with symptom severity [5,57]. Similar phenomena have been observed in patients with CRS, indicating that TRPM8–neuropeptide-mediated neurogenic pathways represent a shared driver of NHR across upper airway inflammatory diseases [5,57].

Animal studies further support this relationship, showing that inflammatory mediator-induced cold allodynia is highly dependent on TRPM8 signaling. In experimental models, TRPM8 knockout mice show abolished cold allodynia induced by endogenous inflammatory mediators [30]. Peripheral injection of CGRP or SP directly induces cold allodynia in mice, an effect absent in TRPM8-deficient animals [17], indicating that CGRP and SP mediate inflammatory mediator-induced cold allodynia and their effects are highly dependent on TRPM8. The use of CGRP receptor antagonists or NK1R antagonists can partially alleviate these responses [17], further confirming the central regulatory role of TRPM8 in cold stimulation-related inflammatory hyperreactivity.

In airway and nasal epithelial cells, cold-activated TRPM8 triggers Ca^2+^ influx and sequentially activates protein kinase C (PKC) and mitogen-activated protein kinase (MAPK)/nuclear factor-κB (NF-κB) signaling pathways [58,59]. Activation of these pathways promotes the release of Th2 cytokines (IL-4, IL-5), pro-inflammatory mediators (IL-6, IL-8, TNF-α), and mucin 5AC (MUC5AC) [60,61]. Consequently, this cascade aggravates airway inflammation, tissue remodeling, and mucus hypersecretion [31]. However, this epithelial TRPM8-driven inflammatory mechanism has not yet been directly validated in AR models, highlighting an important gap for future investigation.

### 3.4. Neuroimmune Mechanisms of TRPM8 in CRS

Recent studies suggest that TRPM8 signaling is also dysregulated in chronic rhinosinusitis. Cai et al. demonstrated through in vivo and in vitro experiments that cinnamaldehyde (CA) alleviates CRS inflammation by targeting TRPM8 and inhibiting the NF-κB pathway [62]. In human nasal epithelial cell (HNEC) models, CA significantly reduced the expression of pro-inflammatory cytokines such as IL-25, IL-33, and TSLP while inhibiting TRPM8 expression and NF-κB p65 phosphorylation. In CRS mouse models, CA reduced nasal scratching and sneezing symptoms, alleviated nasal mucosal inflammation, and decreased the expression of the aforementioned pro-inflammatory cytokines and TRPM8 in nasal lavage fluid and tissues [62]. These findings indicate that synergistic inhibition of TRPM8 and NF-κB signaling contributes to the therapeutic effects of CA in CRS, although the precise upstream–downstream relationships between TRPM8 activation and inflammatory mediator production remain to be clarified.

At the clinical level, Migneault-Bouchard et al. revealed the role of TRPM8 in CRS at the subtype level [57]. Notably, the dominant-negative short isoform sTRPM8-18 is significantly overexpressed in CRS and can bind to the C-terminal domain of full-length TRPM8, thereby suppressing channel activity [57]. This altered expression pattern is hypothesized to impair trigeminal sensory perception of nasal airflow, contributing to the sensation of nasal obstruction commonly reported by CRS patients. These findings link TRPM8 dysregulation not only to inflammatory signaling but also to abnormal sensory perception, reinforcing its relevance to the development and persistence of NHR in CRS.

### 3.5. Functional Divergence and Crosstalk Between TRPA1 and TRPM8

**Figure 2:**
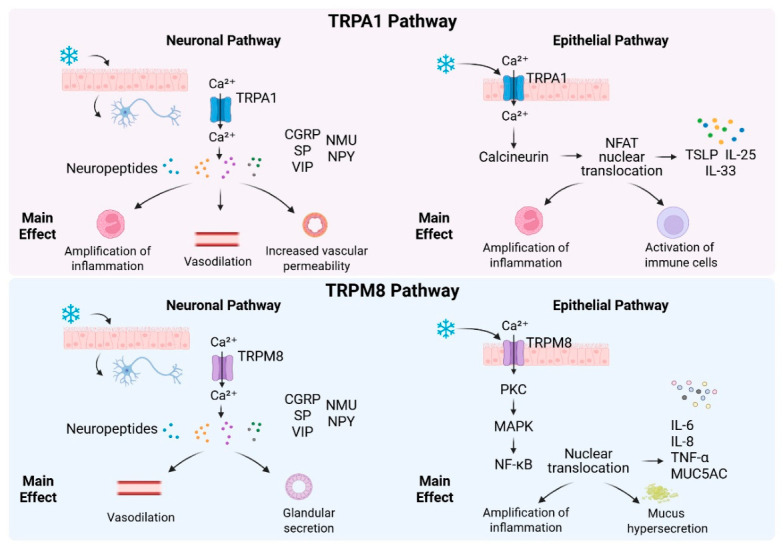
Divergent neuronal and epithelial signaling pathways mediated by TRPA1 and TRPM8 in cold-induced nasal responses. Cold stimulation activates transient receptor potential ankyrin 1 (TRPA1) and transient receptor potential melastatin 8 (TRPM8) expressed on trigeminal sensory neurons and nasal epithelial cells, triggering distinct downstream cascades. In the neuronal pathway, activation of the noxious cold sensor TRPA1 induces robust Ca^2+^ influx and release of neuropeptides, which drive amplification of neurogenic inflammation, vasodilation, and increased vascular permeability. In contrast, activation of the physiological cold sensor TRPM8 primarily promotes vasodilation and glandular secretion without strong pro-inflammatory effects. In the epithelial pathway, TRPA1 triggers Ca^2+^-dependent calcineurin signaling, leading to nuclear factor of activated T cells (NFAT) translocation and production of epithelial alarmins including thymic stromal lymphopoietin (TSLP), interleukin-25 (IL-25), and IL-33. These alarmins activate immune cells and amplify local inflammation. TRPM8-mediated Ca^2+^ signaling preferentially activates protein kinase C (PKC), mitogen-activated protein kinase (MAPK), and nuclear factor kappa B (NF-κB) pathways, driving the production of pro-inflammatory cytokines including IL-6, IL-8, and tumor necrosis factor-α (TNF-α), as well as mucin 5AC (MUC5AC)-dependent mucus hypersecretion. These divergent pathways underlie distinct clinical phenotypes of cold-induced nasal hyperreactivity. (Created in BioRender. Kang, T. (2026) https://BioRender.com/cm0020p, accessed on 26 April 2026).

TRPA1 and TRPM8 exert non-overlapping yet interactive roles in cold-induced nasal hyperreactivity. TRPA1 acts primarily as a pro-inflammatory amplifier via Ca^2+^-NFAT signaling to trigger alarmin release and neurogenic inflammation, whereas TRPM8 functions as a cold sensor regulating trigeminal sensation and mucus secretion through PKC/NF-κB/MAPK pathways [53,58]. Notably, crosstalk occurs in CRS: TRPA1-derived VIP modulates TRPM8-positive nerve terminals to aggravate abnormal airflow perception, and TRPA1 can be aberrantly activated to compensate for impaired TRPM8 function [36,57,62]. This functional duality underlies the phenotypic heterogeneity of cold-related nasal symptoms in AR and CRS. Thus, the distinct neuronal and epithelial signaling programs governed by TRPA1 and TRPM8 collectively shape the heterogeneous phenotypes of cold-induced nasal hyperreactivity (Figure 2).

## 4. Therapeutic Potential of Targeting Cold Thermoreceptors

Given the central role of cold-sensitive TRP channels in the development of nasal hyperreactivity, targeting TRPA1 and TRPM8 represents a promising therapeutic strategy for AR and CRS. In contrast to conventional anti-inflammatory therapies that primarily act on immune effector pathways, modulation of TRPA1 and TRPM8 directly addresses sensory nerve-driven symptom generation and neurogenic inflammation, providing a mechanistically distinct approach with the potential to improve quality of life in patients with refractory NHR.

### 4.1. TRPA1: From Preclinical Evidence to Early Clinical Development

Numerous studies have demonstrated that genetic deletion or pharmacological inhibition of TRPA1 reduces airway inflammation and hyperreactivity in rodent models. For example, the TRPA1 antagonist HC-030031 suppresses TRPA1 expression in the nasal mucosa and vagal ganglia of AR mice, reduces SP release, decreases eosinophil and mast cell infiltration, and downregulates type 2 cytokines such as IL-4 and IL-5. These combined effects alleviate both upper and lower airway inflammation as well as bronchial hyperreactivity [38]. The novel TRPA1 inhibitor GDC-0334 demonstrates high selectivity. In vitro studies show that it inhibits TRPA1 function and airway smooth muscle responses, while animal models confirm its ability to reduce edema, dermal blood flow, cough, and allergic airway inflammation. Phase I clinical trial results indicate that GDC-0334 reduces TRPA1 agonist-induced changes in skin blood flow, pain, and itching in healthy human volunteers, confirming target engagement and a favorable safety profile in humans [27]. Notably, clinical development of GDC-0334 has been terminated (ClinicalTrials.gov: NCT03381144), and no further clinical evaluation has been pursued. This outcome highlights the challenges in translating TRPA1 antagonists from preclinical efficacy to clinical approval. Meanwhile, these findings provide a strong rationale for further clinical evaluation of TRPA1 inhibitors in type 2 inflammatory diseases such as asthma and AR.

### 4.2. TRPM8: Modulation Mechanisms and Therapeutic Explorations

TRPM8 activity can be bidirectionally regulated through multiple mechanisms, including interacting proteins, splice variants, post-translational modifications, specific regulatory regions, and G protein-coupled receptor signaling cascades [63], forming a complex regulatory network that offers multiple opportunities for therapeutic intervention.

TRPM8 activation depends on its interaction with the membrane PI(4,5)P_2_. Sustained cold stimulation induces Ca^2+^ influx and activates the phospholipase C (PLC)–PKC pathway, leading to PI(4,5)P_2_ depletion and TRPM8 phosphorylation, thereby promoting channel desensitization [24,63,64,65]. This negative regulation of channel function prevents tissue damage caused by excessive cold stimulation.

The TRPM8 antagonist N-(4-tert-butylphenyl)-4-(3-chloropyridin-2-yl)piperazine-1-carboxamide (BCTC) blocks the TRPM8-Ca^2+^-PKC axis-promoted synthesis and secretion of MUC5AC, alleviating rhinitis symptoms by targeting the pathological mechanism [31]. Furthermore, cinnamaldehyde has been verified to alleviate CRS inflammation by inhibiting TRPM8 and the NF-κB pathway [62]. Evidence from neurological disease models further suggests that TRPM8 antagonists protect against cold-induced Ca^2+^ influx and aberrant TRPM8 expression in the sciatic nerve, offering potential therapeutic avenues for CRS-associated pain and sensory dysfunction [23].

### 4.3. Current Limitations of TRP Channel-Targeted Therapy for AR and CRS

To date, no TRPA1 or TRPM8 modulators have been approved for clinical use in AR or CRS, with most interventions remaining in preclinical or early-phase clinical stages [22,23]. TRPA1 antagonists have reached Phase I trials for pain and airway disorders, but development for upper airway diseases has been discontinued, while TRPM8-targeted agents are still restricted to preclinical research [13,27]. A major translational barrier is the inherent limitation of preclinical animal models: rodent models fail to fully recapitulate the human nasal anatomical complexity, chronic inflammatory microenvironment, comorbidity profiles, and long-term disease progression of AR and CRS [22]. Moreover, species differences in TRP channel expression patterns, thermal activation thresholds, and neuroimmune coupling mechanisms between rodents and humans lead to inconsistent translation of preclinical efficacy to clinical outcomes [12,21]. Additional potential limitations include poor subtype selectivity of early modulators, which may cause off-target effects; heterogeneous expression of TRP splice variants that leads to variable patient responsiveness; and a lack of studies focusing on long-term disease modification rather than acute symptomatic relief [23,57,58]. These unmet gaps underscore the urgency of developing clinically relevant human ex vivo models and conducting large-scale, longitudinal clinical trials to enhance the translational robustness of TRP channel-targeted therapies.

Collectively, these findings underscore the therapeutic promise of cold-sensitive receptors as drug targets. Future research should prioritize elucidating TRPM8 post-translational regulation and signaling interactions to optimize antagonist selectivity, efficacy, and safety, thereby advancing precision medicine approaches for AR and CRS.

## 5. Conclusions

In summary, TRPA1 and TRPM8 function as key cold-sensitive receptors that integrate environmental cold stimuli with sensory nerve activation, neuroimmune inflammation, and nasal hyperreactivity in AR and CRS. Specifically, TRPA1 mainly acts as a pro-inflammatory amplifier, while TRPM8 primarily serves as a cold-sensory and patency regulator with distinct pathological contributions. Activation of these channels induces neurogenic inflammation through neuropeptide release and immune cell activation, contributing to persistent and refractory nasal symptoms. Conceptualizing NHR as a neuroimmune disease phenotype provides a unifying framework for understanding symptoms that cannot be fully explained by classical immune mechanisms alone. Most clinical trials of TRPA1 and TRPM8 modulators have focused on sensory pain, neurological disorders, and dermatological conditions, with scarce direct clinical evidence supporting their use in AR and CRS. Translational gaps in preclinical animal models and developmental constraints of TRP-targeted interventions further impede clinical application. This collective evidence underscores an urgent need for dedicated clinical trials evaluating TRP channel-targeted strategies for NHR in these upper airway diseases. Although clinical interventions directly targeting TRPA1 and TRPM8 remain limited, ongoing advances in understanding TRP channel isoform diversity, context-dependent activation, and neuroimmune signaling networks hold promise for the development of individualized precision therapies. Through systematic dissection of these molecular pathways, it is anticipated that targeted modulation of sensory–immune interactions will enhance therapeutic efficacy and improve quality of life for patients with AR and CRS.

## Data Availability

No new data were created or analyzed in this study.

## Abbreviations

The following abbreviations are used in this manuscript:

AR	Allergic rhinitis
BCTC	N-(4-tert-butylphenyl)-4-(3-chloropyridin-2-yl) piperazine-1-carboxamide
CA	Cinnamaldehyde
cAMP	Cyclic adenosine monophosphate
CDA	Cold-dry air
CGRP	Calcitonin gene-related peptide
CLR	Calcitonin receptor-like receptor
CNS	Central nervous system
CRS	Chronic rhinosinusitis
HNEC	Human nasal epithelial cell
IL	Interleukin
ILC2s	Type 2 innate lymphoid cells
MAPK	Mitogen-activated protein kinase
MC	Mast cell
MCs	Mast cells
MUC5AC	Mucin 5AC
NFAT	Nuclear factor of activated T cells
NF-κB	Nuclear factor-κB
NHR	Nasal hyperreactivity
NK1R	Neurokinin 1 receptor
NMU	Neuromedin U
NPY	Neuropeptide Y
NLRP3	NOD-like receptor family pyrin domain-containing 3
P-GATA3	Phosphorylated GATA-binding protein 3
PI(4,5)P2	Phosphatidylinositol 4,5-bisphosphate
PKA	Protein kinase A
PKC	Protein kinase C
PLC	Phospholipase C
PNIF	Peak nasal inspiratory flow
SP	Substance P
TLR	Toll-like receptor
TNF-α	Tumor necrosis factor-α
TNC	Trigeminal nucleus caudalis
TRP	Transient receptor potential
thermoTRP	Thermosensitive transient receptor potential channel
TRPA1	Transient receptor potential ankyrin 1
TRPM8	Transient receptor potential melastatin 8
sTRPM8-18	Short isoform of TRPM8-18
TRPV1	Transient receptor potential vanilloid 1
TSLP	Thymic stromal lymphopoietin
VIP	Vasoactive intestinal peptide
VPAC	Vasoactive intestinal peptide receptor
Y1R	Neuropeptide Y receptor 1
Y2R	Neuropeptide Y receptor 2
